# Vegetation-Mediated Soil Organic Carbon Differentiation and Carbon Sequestration Strategies in a Typical Wetland of the North China Plain

**DOI:** 10.3390/plants15101524

**Published:** 2026-05-16

**Authors:** Zonglin Shi, Yan Wang, Xiaoshuang Li, Na Zhang, Sisi Li, Yue Wang, Hongjun Lin, Yuhong Dong, Hongju Zhou, Dayong Wu, Man Cheng

**Affiliations:** 1 College of Life Science, Hengshui University, Hengshui 053000, China; lxsh1980@126.com (X.L.); li_sisi_2010@126.com (S.L.); jyailn@163.com (Y.W.); dongyangsea@163.com (H.L.); 694396694@163.com (Y.D.); jvzhouhong@163.com (H.Z.); 2 School of Geography and Tourism, Shaanxi Normal University, Xi’an 710119, China; 15294427978@163.com; 3 Hebei Key Laboratory of Wetland Ecology and Conservation, Hengshui University, Hengshui 053000, China; zhangna118wood@163.com (N.Z.); dayongwu@hotmail.com (D.W.); 4 School of Environmental and Resource Sciences, Shanxi University, Taiyuan 030006, China

**Keywords:** wetland soil, vegetation type, soil organic carbon fraction, soil aggregate, microbial community

## Abstract

Soil organic carbon (SOC) responds rapidly to vegetation changes, and exploring SOC sequestration mechanisms under different vegetation types is critical for optimizing wetland carbon sink functions. This study investigated the abiotic and biotic mechanisms driving SOC stability across four typical vegetation types (reed marsh, woodland, farmland, and wasteland) in the 0–10 cm and 10–20 cm soil layers of Hengshui Lake wetland. Results showed that reed marshes exhibited the highest total organic carbon (TOC) and particulate organic carbon (POC), owing to anaerobic soil conditions and stable macroaggregate physical protection. Woodlands accumulated higher dissolved organic carbon (DOC) and microbial biomass carbon (MBC) via an efficient microbial carbon pump, despite weaker aggregate stability. In contrast, farmlands and wastelands presented intense labile organic carbon (LOC) turnover and enzymatic decomposition, accelerating SOC mineralization and carbon dissipation with poor carbon sequestration capacity. Proteobacteria and Acidobacteriota dominated bacterial communities, while Ascomycota prevailed in fungi. Soil water content (SWC) and bulk density (BD) were the core drivers of microbial community succession, and fungi were more sensitive to vegetation changes. Conclusively, distinct vegetation types shape divergent SOC sequestration pathways. This work provides a theoretical basis for wetland restoration and regional carbon sink enhancement.

## 1. Introduction

Wetlands represent one of the most productive ecosystems on Earth and are widely acknowledged as a cornerstone of natural climate solutions for mitigating climate change, serving a central function in the global terrestrial carbon cycle [1]. Although covering only about 8% of the global land surface, wetlands store an estimated 29% to 45% of terrestrial organic carbon, making them disproportionately important in the global carbon cycle [2,3,4,5]. Lake wetlands, as critical aquatic–terrestrial transition zones, play an irreplaceable role in regional climate regulation, water purification and long-term carbon sequestration [4]. However, under the combined pressure of climate change and anthropogenic disturbance, more than half of the world’s wetlands have experienced severe degradation, threatening the stability of their carbon source and sink functions [6]. Even subtle fluctuations in wetland soil carbon pools can trigger substantial changes in atmospheric CO_2_ concentrations and amplify climate feedback loops [7], rendering the exploration of wetland soil organic carbon (SOC) stability mechanisms essential for advancing the understanding of wetland carbon cycle responses to global change and informing climate mitigation strategies.

Previous studies have elaborated SOC accumulation mechanisms and the relationships between SOC dynamics and soil physicochemical and biological properties, forming a relatively complete theoretical framework for soil organic carbon cycling [5,8,9,10]. At the macro scale, vegetation type and climatic conditions have been confirmed as dominant drivers controlling the vertical distribution of total organic carbon (TOC) and its functional fractions [11,12]. In this context, ecological restoration measures, including wetland rehabilitation and afforestation, have been proven effective in restoring depleted SOC stocks and offsetting more than half of carbon emissions derived from wetland degradation [13]. Nevertheless, wetland structural and functional recovery is a complex process comprehensively constrained by soil physical properties, nutrient availability, natural disturbances, and biodiversity. Notably, plant functional traits exhibit divergent regulatory pathways for soil carbon pool dynamics [7,14]. Succession ecological theory further highlights that vegetation restoration enhances soil carbon sinks not only through increasing litter inputs but also by reshaping soil microbial communities, which in turn promote long-term carbon stabilization via physicochemical interactions [15]. Thus, clarifying the regulatory mechanisms underlying SOC storage and stability under vegetation restoration is a core scientific prerequisite for accurately evaluating wetland restoration efficacy and formulating targeted carbon sink enhancement strategies.

Accurate assessment of SOC sequestration capacity and stability requires characterization of functionally distinct carbon fractions rather than reliance on total organic carbon (TOC) alone [16]. Particulate organic carbon (POC), derived mainly from partially decomposed plant detritus, is highly responsive to physical protection within soil aggregates [17]. Dissolved organic carbon (DOC) and microbial biomass carbon (MBC) reflect microbial assimilation and biogeochemical transformation efficiency [18], whereas labile organic carbon (LOC) serves as a sensitive indicator of carbon lability and environmental conditions [19]. Current consensus has confirmed that plants regulate carbon inputs via root morphology and exudation, creating divergent pathways for carbon stabilization [20]. The Microbial Efficiency-Matrix Stabilization (MEMS) framework further posits that the quality of litter and rhizodeposits determines microbial carbon use efficiency, driving the conversion of labile plant-derived carbon into stable microbial necromass carbon [21,22]. Concurrently, root exudates serve as binding agents to facilitate macroaggregate formation, and microscale physical occlusion protects organic metabolites from enzymatic decay, creating a coupled system of short-term biotransformation and long-term physical stabilization [23]. Despite substantial progress in understanding individual carbon sequestration pathways, most existing studies decouple aggregate physical protection from microbial community functions dynamics and ignore their internal linkage. Soil aggregates function as key microhabitats that maintain intensive interactions between labile substrates (DOC and LOC) and microbial biomass carbon (MBC) [24,25], thereby driving the turnover and stabilization of TOC. Such chain-like interactions mediated by aggregates are often overlooked in fragmented studies [26]. More importantly, carbon turnover balance, regulated by microhabitat properties, undergoes drastic pathway shifts under complex environmental gradients, especially in inland salinized wetlands. In such unique habitats, Salinity not only acts as an environmental filter to impose physiological stress on microbial communities but also induces soil clay flocculation and aggregate formation, reducing substrate accessibility and inhibiting soil organic matter decomposition via physical shielding effects [27]. This salinity-mediated “physical-biological coupled carbon sequestration mechanism” has become a research frontier in biogeochemistry. However, it remains poorly understood how multiple physicochemical stressors and microbial metabolic pathways jointly modulate the differentiation of SOC functional fractions remains poorly understood. This critical limitation restricts the mechanistic cognition of carbon cycling in salinized inland wetlands and hinders the optimization of targeted wetland carbon sink enhancement strategies.

Hengshui Lake Wetland is the only nature reserve in the North China Plain preserving an intact wetland ecosystem, including marshes, open water, tidal flats, meadows and forests. As a key ecological node supporting the Xiong’an New Area ecological barrier, it exerts a strong regulatory effect on the Beijing–Tianjin–Hebei regional climate and represents a model inland freshwater wetland ecosystem [28]. Historically, the area has undergone cycles of agricultural reclamation and wetland restoration. In recent years, large-scale ecological restoration practices, including farmland-to-wetland and farmland-to-forest/grassland conversions, have been implemented under national ecological policies [29]. Dynamic changes in land-use and vegetation restoration inevitably alter soil properties and microbial community structure, thereby reshaping local SOC cycling processes [4,13,15,30]. Distinct from conventional freshwater and coastal wetlands, Hengshui Lake wetland features a unique habitat combining freshwater background and mild soil salinization [29], and its carbon cycle process exhibits higher spatial heterogeneity and mechanistic complexity than those of typical wetland ecosystems. Against the backdrop of China’s national policies to advance “carbon peek” and “carbon neutrality”, this study aims to: (i) characterize the vertical distribution of TOC and its fractions under different vegetation types; (ii) reveal the coupling relationships among soil physicochemical properties, extracellular enzyme activities, aggregate stability, microbial communities, and carbon fraction accumulation; (iii) clarify the differential regulatory mechanisms of carbon sequestration during vegetation succession, and proposing corresponding regulatory strategies. We hypothesized that different vegetation types shape distinct SOC sequestration pathways via divergent physical structure protection and microbial regulatory strategies, resulting in significant differences in SOC fraction accumulation and stability. This study is expected to provide theoretical support for the optimization of vegetation types and carbon sink enhancement management in the ecological restoration of Hengshui Lake Wetland, offer a scientific reference for the rehabilitation of similar inland salinized wetlands in the North China Plain, and also enrich the global wetland carbon cycle theory system while providing a universal reference for carbon sink management of analogous inland wetland ecosystems worldwide to support global climate change mitigation and carbon neutrality efforts.

## 2. Results

### 2.1. SOC Fractions Distribution

Vegetation type significantly affected SOC fractions (TOC, POC, LOC, DOC, and MBC) (*p* < 0.05), with concentrations generally decreasing with increasing soil depth (Table 1). In the 0–10 cm soil layer, TOC (15.92 g kg^−1^) and POC (6.65 g kg^−1^) were highest in reed marsh, while TOC was lowest in farmland (7.41 g kg^−1^) and POC in wasteland (1.71 g kg^−1^). DOC (239.75 mg kg^−1^) and MBC (558.28 mg kg^−1^) peaked in woodland, with DOC lowest in reed marsh (85.89 mg kg^−1^) and MBC in wasteland (215.93 mg kg^−1^). LOC was the highest in farmland (3.66 g kg^−1^) and lowest in woodland (1.45 g kg^−1^). SOC fractions variation patterns among different vegetation types in the 10–20 cm soil layer were generally consistent with the 0–10 cm layer.

### 2.2. Soil Physicochemical Properties and Enzyme Activities

As shown in Table 2, vegetation types significantly affected soil physicochemical properties (except for pH) (*p* < 0.05). In the 0–10 cm layer, reed marsh had the highest soil water content (SWC, 44.93%), electrical conductivity (EC, 462.33 μs cm^−1^), and total nitrogen (TN, 1.30 g kg^−1^), along with the lowest bulk density (BD, g cm^−3^). Farmland exhibited the highest BD (1.58 g cm^−3^), pH (8.83), and available phosphorus (AP, 12.63 mg kg^−1^), but the lowest EC (91.91 μs cm^−1^). Wasteland had the highest total phosphorus (TP, 1.09 mg kg^−1^) and total potassium (TK, 17.95 mg kg^−1^), with the lowest pH (8.83). Woodland displayed the lowest water content (13.43%) and nutrient levels, with AP, TP, TN and TK all significantly lower than in other vegetation types (*p* < 0.05). In the 10–20 cm layer, variations in most parameters were generally consistent with the surface layer, although differences in some indices diminished.

All soil urease (URE), alkaline phosphatase (ALP), sucrase (SUC), protease (PRO), and catalase (CAT) activities differed significantly among the four vegetation types (*p* < 0.05) (Table 3). In the 0–10 cm layer, wasteland had the highest URE (1.31 mg g^−1^ d^−1^) and ALP (1.74 mg g^−1^ d^−1^) activities, farmland the highest CAT (2.29 mL g^−1^ 20 min^−1^), woodland the highest PRO (0.24 mg g^−1^ d^−1^), and reed marsh the highest SUC (35.92 mg g^−1^ d^−1^). Except for PRO, the other four enzyme activities were lowest in the woodland. In the 10–20 cm layer, CAT, PRO, and ALP activity trends were consistent with the 0–10 cm layer, while URE (1.03 mg g^−1^ d^−1^) and SUC (19.24 mg g^−1^ d^−1^) activities peaked in wasteland. Most soil enzyme activities decreased with soil depth.

### 2.3. Soil Aggregate Distribution and SOC in Aggregate

**Figure 1:**
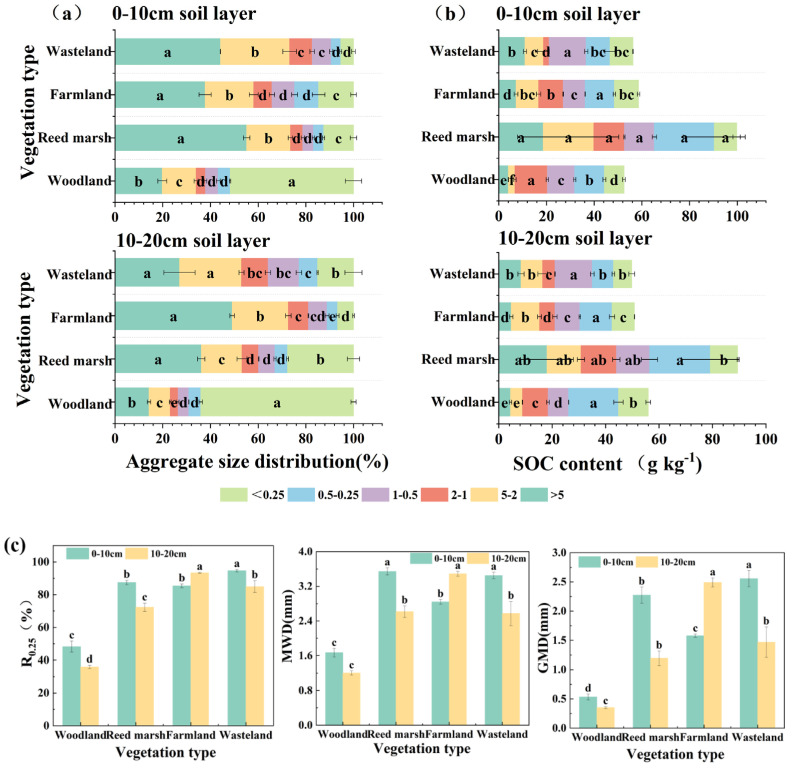
Soil aggregate distribution and SOC in aggregates under different vegetation types. Changes in soil aggregate distribution (**a**) and SOC in aggregates (**b**) at 0–10 cm and 10–20 cm soil layers, respectively. (**c**) Changes in soil aggregate stability indices; different lowercase letters indicate significant differences among vegetation types. R_0.25_, proportion of aggregates >0.25 mm; MWD, mean weight diameter; GMD, geometric mean diameter. Different lowercase letters indicate significant differences among aggregate size classes within the same vegetation type (*p* < 0.05).

Soil aggregate distribution differed significantly among vegetation types (*p* < 0.05) (Figure 1a). In the 0–10 cm soil layer, soil aggregates were predominantly with <0.25 mm in woodland (51.74%), >5 mm in size in reed marsh (55.20%), wasteland (44.20%), and farmland (37.78%). Although aggregate size fluctuated slightly among vegetation types in the 10–20 cm soil layer, the overall trend was consistent with the 0–10 cm layer. Under all vegetation types, SOC content was highest in 0.25–2 mm (0–10 cm) and 0.25–1 mm (10–20 cm) aggregates; the reed marsh generally exhibited the highest SOC content within the aggregate, with no significant differences among aggregate sizes in the 0–10 cm layer (*p* > 0.05) (Figure 1b). As shown in Figure 1c, vegetation types significantly influenced soil aggregate stability (*p* < 0.05). In the 0–10 cm layer, macroaggregates dominated in wasteland, farmland, and reed marsh (R_0.25_ remained above 85.00% for all), with the lowest proportion in woodland (Figure 1c). Wasteland had the highest aggregate stability, with proportion of aggregates >0.25 mm (R_0.25_), mean weight diameter (MWD), and geometric mean diameter (GMD) significantly higher than the other three vegetation types (*p* < 0.05). In the 10–20 cm layer, farmland had the significantly highest aggregate stability, which increased with soil depth.

### 2.4. Bacterial and Fungal Community Composition and Diversity

**Figure 2:**
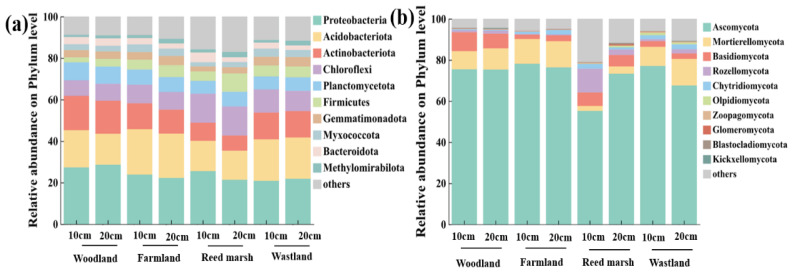
Microbial community composition at the phylum level. (**a**) Relative abundance of bacterial communities; (**b**) relative abundance of fungal communities. Different colors represent different microbial phyla. “10 cm” and “20 cm” indicate the 0–10 cm and 10–20 cm soil layers, respectively.

At the phylum level, the dominant soil bacterial phyla were Proteobacteria (20.86–28.67%), Acidobacteriota (14.0–21.87%), Actinobacteriota (7.27–16.52%), Chloroflexi (7.51–14.01%) and Planctomycetota (6.17–8.65%) (Figure 2a), collectively accounting for more than 70.00% of the total bacterial sequence. Specifically, Proteobacteria, Actinobacteriota, and Planctomycetota were more abundant in woodland soils, Acidobacteriota in farmland and wasteland soils, and Chloroflexi in reed marsh. For fungal (Figure 2b), Ascomycota was the absolutely dominant phylum (55.21–78.14%), followed by Mortierellomycota (2.42–12.95%) and Basidiomycota (1.21–9.19%). Fungal community structure was similar across woodland, farmland, and wasteland, but in the 0–10 cm surface soil of reed marsh, Ascomycota abundance decreased to 55.21%, while Rozellomycota increased to 11.57%, higher than Mortierellomycota (2.41%) and Basidiomycota (6.53%). At 10–20 cm, Ascomycota abundance recovered, and fungal communities converged with other samples. Under all vegetation types, microbial phylum-level composition was consistent across soil layers, indicating soil depth had a weaker effect than vegetation type, which was the core driver of microbial community differentiation.

**Figure 3:**
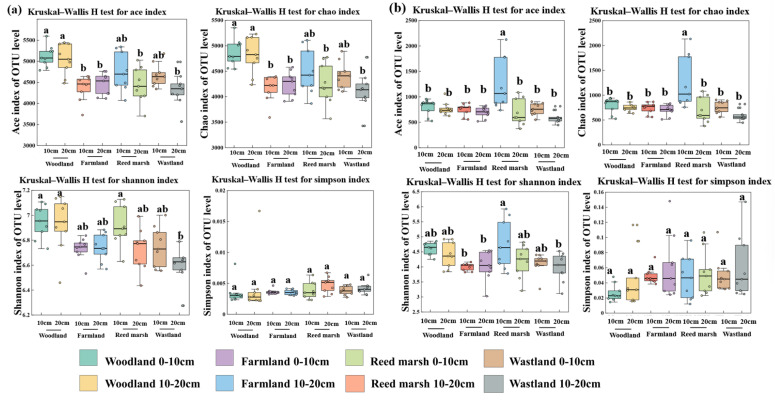
Soil microbial community alpha diversity indices. (**a**) Ace, Chao1, Shannon and Simpson indices of bacteria; (**b**) Ace, Chao1, Shannon and Simpson indices of fungi. Different lowercase letters indicate significant differences among all soil samples (*p* < 0.01).

Alpha diversity of soil bacteria and fungi was evaluated using richness (Ace, Chao) and diversity indices (Shannon, Simpson), with significant variations among vegetation types in the 0–20 cm soil layer (Figure 3). For bacteria (Figure 3a), woodland soils had the highest richness and diversity across depths. Specifically, the Ace and Chao indices in woodland soils were significantly higher than those in farmland and wasteland soils (10–20 cm layer), and the Shannon index was significantly greater than that in wasteland (10–20 cm layer) (*p* < 0.01). No significant difference in Simpson index was observed across all soil samples (*p* > 0.01), and the alpha diversity of bacteria showed no significant downward trend with soil depth (*p* > 0.01). For fungi (Figure 3b), Ace and Chao indices in the 0–10 cm soil layer of reed marsh were significantly higher than those in other samples (*p* < 0.01), and the Shannon index was significantly greater than that in farmland (0–20 cm) and wasteland (10–20 cm). Simpson index showed no intergroup differences (*p* > 0.05). Fungal alpha diversity decreased with soil depth across all samples, with an extremely significant reduction in reed marsh (*p* < 0.01). These results confirm vegetation type as the dominant factor regulating soil bacterial and fungal alpha diversity.

**Figure 4:**
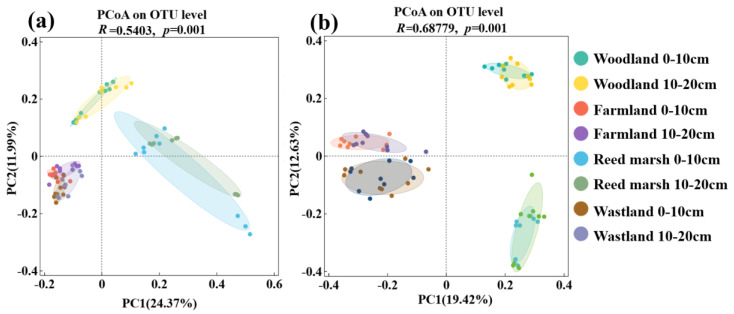
Principal Coordinate Analysis (PCoA) of soil bacterial (**a**) and fungal (**b**) community structures based on Bray–Curtis distance at the OUT level. Dots of different colors represent different treatments from four vegetation types (woodland, reed marsh, farmland, and wasteland) at two soil depths (0–10 cm and 10–20 cm). Analysis of similarities (ANOSIM) was performed to test significant differences among treatments. Each treatment included 9 microbial samples (3 biological replicates × 3 technical replicates).

Principal Coordinate Analysis (PCoA) based on Bray–Curtis distance and ANOSIM tests revealed that vegetation type significantly altered soil bacteria (R = 0.5403, *p* = 0.001) and fungi (R = 0.6878, *p* = 0.001) community structure, with greater inter-group differentiation observed in fungal communities than in bacterial communities (Figure 4). For the bacterial community (Figure 4a), the first two axes explained a cumulative 36.36% of the total variation (PC1 = 24.37%, PC2 = 11.99%). Spatially, bacterial communities from the four vegetation types formed three distinct clusters: reed marsh samples were aggregated in the positive direction of PC1, woodland samples were concentrated in the positive direction of PC2, while farmland and wasteland samples were distributed in the negative direction of both PC1 and PC2. Notably, the 95% confidence ellipses of farmland and wasteland partially overlapped, suggesting a certain degree of similarity in bacterial community structure between these two vegetation types. For the fungal community (Figure 4b), the first two axes cumulatively explained 32.05% of the total variation (PC1 = 19.42%, PC2 = 12.63%) with woodland in the positive direction of PC1 and PCA2, farmland in the negative direction of PCA1 and positive direction of PCA2, reed marsh in the positive direction of PCA1 and negative direction of PCA2, and wasteland in the negative direction of PCA1 and PCA2, which formed four independent and well-defined clusters along PC1 and PC2. Furthermore, for both bacteria and fungi communities, the 95% confidence ellipses of samples from 0 to 10 cm and 10–20 cm soil layers overlapped substantially within each vegetation type, indicating consistent variation across soil depth. These results confirm that the distribution of bacteria and fungi communities was mainly driven by vegetation type, whereas soil depth exerted a comparatively weak influence.

### 2.5. Coupling Relationships Among Soil Physicochemical Properties, Aggregate Stability, Enzyme Activities, Microbial Communities, and SOC Fractions Differentiation

**Figure 5:**
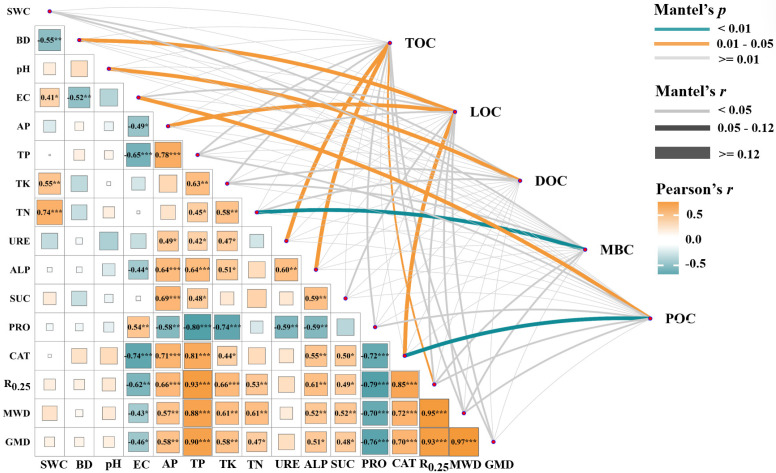
Coupling relationship network of soil environmental factors and SOC fractions based on Pearson correlation analysis and pairwise Mantel tests. The lower-left heatmap shows Pearson correlation coefficients among soil physicochemical properties (SWC, BD, pH, EC, AP, TP, TK, TN), enzyme activities (URE, ALP, SUC, PRO, CAT), and aggregate stability indices (R_0.25_, MWD, GMD). Color intensity and hue indicate the strength and direction of correlations (orange: positive; blue: negative; * *p* < 0.05, ** *p* < 0.01, *** *p* < 0.001). The upper-right network illustrates Mantel test results for the coupling relationships between soil attributes and SOC fractions (TOC, POC, LOC, DOC, MBC). Line color indicates significance (cyan: *p* < 0.01; orange: 0.01 ≤ *p* < 0.05; gray: *p* ≥ 0.05), and line width represents the absolute value of Mantel’s r (wider lines denote stronger correlations). SWC, soil water content; BD, bulk density; EC, electrical conductivity; TP, total phosphorus; AP, available phosphorus; TK, total potassium; TN, total nitrogen; URE, soil urease activity; ALP, alkaline phosphatase activity; SUC, sucrase activity, PRO, protease activity; CAT, catalase activity; R_0.25_, proportion of aggregates >0.25 mm; MWD, mean weight diameter; GMD, geometric mean diameter.

Pearson correlation analysis revealed strong synergistic changes among soil environmental factors (Figure 5). Specifically, EC exhibited highly significant negative correlations (*p* < 0.001) with soil aggregate stability indices (R_0.25_, MWD and GMD), while TP showed highly significant positive correlations (*p* < 0.001) with these indices, indicating a distinct positive synergistic effect. For enzyme activities, CAT was highly significantly positively correlated with R_0.25_ and MWD (*p* < 0.001), whereas PRO showed highly significant negative correlation (*p* < 0.001) with aggregate stability indices and key nutrients such as TP and TK, confirming close linkages between soil enzyme activities, soil structure, and nutrient status. Mantel tests (with 999 permutations) demonstrated significant differences in the key driving factors of different SOC fractions. TOC was significantly correlated with R_0.25_ (r = 0.105, *p* = 0.049), URE (r = 0.147, *p* = 0.028), and ALP (r = 0.122, *p* = 0.049). LOC was co-driven by multiple environmental factors with strong correlations, including BD (r = 0.132, *p* = 0.034), AP (r = 0.129, *p* = 0.017), and CAT (r = 0.130, *p* = 0.024). DOC was only significantly correlated with pH (r = 0.177, *p* = 0.011), indicating a relatively simple driving mechanism. MBC was exclusively highly significantly correlated with TN (r = 0.186, *p* = 0.002), identifying TN as the core factor regulating MBC variation. POC showed the strongest and most stable correlations with EC (r = 0.167, *p* = 0.019) and CAT (r = 0.190, *p* = 0.008). Overall, soil aggregate structure, nutrient contents, and enzyme activities collectively drove the spatial variation in SOC fractions, with MBC and POC being the most sensitive to environmental changes.

**Figure 6:**
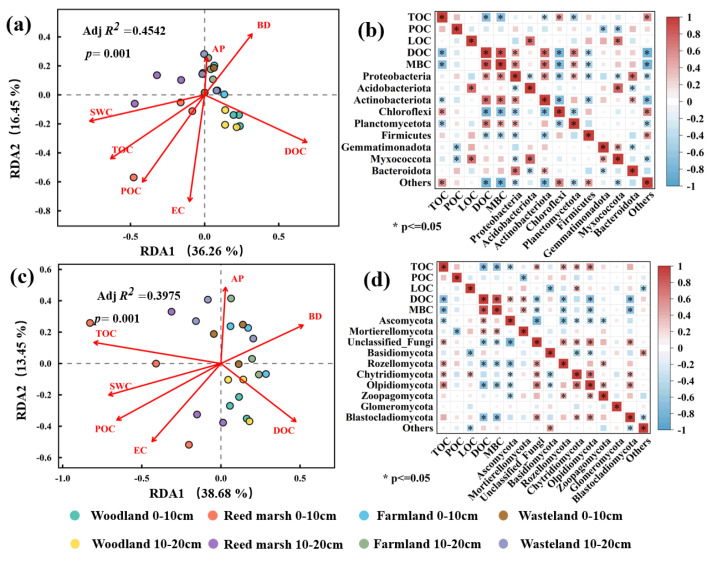
Redundancy analysis (RDA) of soil bacterial (**a**) and fungal (**c**) phylum-level community structure constrained by key environmental factors, and Spearman’s rank correlation heatmaps between dominant bacterial (**b**) and fungal (**d**) phyla and SOC fractions. Dots of different colors represent different treatments from four vegetation types (woodland, reed marsh, farmland, and wasteland) at two soil depths (0–10 cm and 10–20 cm). To reduce technical variation, microbial community data were averaged across technical replicates within each biological replicate; thus, each treatment contained three biological replicates for RDA. The adjusted explanatory power (Adj *R*^2^) and overall model significance (*p*-value) were determined using Monte Carlo permutation tests with 999 permutations. Key environmental factors were retained after variance inflation factor (VIF < 10) screening. The “unclassified fungal phylum” observed in the correlation heatmap was aggregated into the “Others” category in the community composition plot for visualization clarity. An asterisk (*) indicates significance at the 0.05 level. The color gradient (from red to blue) represents correlation intensity; red indicates a positive correlation, while blue indicates a negative correlation.

Redundancy analysis (RDA) and Spearman correlation heatmaps (Figure 6) identified directional effects of soil physicochemical factors on microbial community succession and SOC fraction differentiation, with all RDA models highly significant (*p* = 0.001). For bacterial communities (Figure 6a,b), the RDA model explained 45.42% of total variation (adjusted *R*^2^ = 0.4542), with the first two axes accounting for 52.71% of the variation (RDA1 = 36.26%, RDA2 = 16.45%). SWC, TOC, and POC structured reed marsh bacterial communities, enriching Chloroflexi (positively correlated with TOC, negatively with labile fractions DOC/MBC). BD shaped farmland and wasteland communities favoring Acidobacteriota (positively correlated with LOC). DOC drove woodland bacterial communities, selecting for Proteobacteria, Actinobacteriota, and Planctomycetota (positively correlated with DOC/MBC, negatively with TOC). Fungal communities showed consistent environmental responses (Figure 6c,d), with the RDA model explaining 39.76% of variation (adjusted *R*^2^ = 0.3976) and the first two axes accounting for 52.13% of total variation (RDA1 = 38.68%, RDA2 = 13.45%). SWC synergized with POC/TOC to structure reed marsh fungal communities, enriching Rozellomycota (positively correlated with TOC, negatively with MBC/DOC). DOC drove woodland fungal communities, favoring Mortierellomycota (positively correlated with MBC, negatively with POC) and Basidiomycota (negatively correlated with LOC). BD and AP structured farmland and wasteland fungal communities, where dominant Ascomycota were strongly positively correlated with DOC and significantly negatively correlated with TOC (*p* < 0.05). Unclassified fungi were positively correlated with TOC and negatively with MBC/DOC, underscoring their key role in carbon sequestration.

## 3. Discussion

### 3.1. Regulatory Mechanisms of Soil Physicochemical Properties on SOC Fractions

SOC pools are functionally classified into labile, slow, and recalcitrant fractions by turnover time. The labile fraction, including DOC, MBC, LOC, POC, is characterized by rapid turnover, low stability, and high susceptibility to oxidative mineralization [31]. Despite accounting for a small proportion of total SOC, this fraction exhibits exceptional sensitivity to the environmental perturbations, making it a robust indicator of SOC pool dynamics and a potential proxy for global climate change responses [32]. Previous studies have demonstrated that the accumulation and fractionation of SOC in wetlands are governed by biotic and abiotic factors, including wetland type, hydrological regimes, vegetation type, land use type, soil nutrients, pH, salinity, etc. [33]. Among these, vegetation succession acts as a pivotal biotic regulator by reshaping soil physicochemical properties such as SWC, salinity, and BD, and redirecting SOC partitioning among functional fractions. This regulatory mechanism is fully verified by our four vegetation types comparison, also consistent with reservoir wetland research linking vegetation succession to altered soil carbon distribution and sequestration potential [34]. In natural wetland ecosystems, the classic “enzymic latch” theory attributes soil carbon preservation to waterlogged anaerobic conditions, which constrain hydrolase activity and thereby reduce organic carbon decomposition [7,35,36]. Consistent with this consensus, the reed marsh topsoil in this study exhibited high SWC (44.93%) and low BD, forming a stable anaerobic microenvironment. Correspondingly, it had significantly higher TOC (15.92 g kg^−1^) and POC (6.65 g kg^−1^) contents than woodland, wasteland, and farmland. This finding further confirms the core protective effect of anaerobic microhabitats on SOC sequestration in wetlands. However, at the microscopic driving mechanism level, our results differ distinctly from the traditional Microbial-Enzyme Model. This classic model proposes that high biomass input induces elevated hydrolase activity, thereby accelerating soil organic carbon decomposition [31,37]. Mantel test in this study showed that the spatial variation in POC in reed marsh habitats was not regulated by hydrolases (PRO and SUC, *p* > 0.05), but was significantly driven by CAT (*p* < 0.01) and EC (*p* < 0.05). This distinct contrast provides habitat-specific evidence that saline stress (represented by EC) and unique redox conditions (represented by CAT) exert an overriding effect on organic carbon turnover in inland saline wetlands [38,39]. Likewise, studies on saline–alkali wetlands in the Yellow River Delta have also confirmed that salinity acts as a core driver governing wetland SOC turnover [40]. In exploring the stability mechanisms of soil organic carbon (SOC), the traditional view emphasizes the core roles of intrinsic recalcitrance and humic substances (HSs) [41,42]. Gerke highlighted that humic polymers possess strong inherent degradation resistance and can stabilize labile organic molecules (e.g., amino acids) through chemical bonding, thereby substantially reducing their turnover rates [43]. Furthermore, recent high-resolution molecular characterization has confirmed that natural non-pyrogenic humification processes can facilitate the large-scale formation of condensed aromatic macromolecules with intrinsic chemical recalcitrance from biomass [44]. In contrast, an emerging paradigm argues that the long-term persistence of soil carbon is not determined by the intrinsic structural complexity of organic molecules but is primarily driven by spatial inaccessibility and physical environmental constraints. This theory proposes that organic carbon can be physically protected via aggregate encapsulation or mineral surface adsorption, which isolates organic substrates from microbial and extracellular enzyme access and consequently sustains long-term soil carbon preservation [9,45]. Integrating the above two conceptual frameworks, we propose that soil carbon stability results from the synergistic interaction between intrinsic chemical properties and physical spatial constraints, whereby one mechanism may dominate the carbon stabilization process under specific habitat conditions. Numerous studies have demonstrated that, relative to natural wetlands, long-term mechanical tillage in farmland disrupts soil aggregate structure, weakens physical protection, increases soil aeration and organic substrate accessibility, and consequently accelerates the SOC decomposition and loss [31,46,47]. Our farmland soils had higher LOC content but lower TOC content. Owing to high BD and low SWC, the anaerobic protective environment was destroyed, which accelerated the mineralization and depletion of LOC, and consequently reduced TOC and destabilized the soil carbon pool [46]. More notably, traditional succession theory generally suggests that soil carbon recovery can be rapidly initiated after farmland abandonment [15]. However, global meta-analytical evidence has demonstrated that soil ecosystems commonly experience net carbon loss during the initial 0–5 years following agricultural abandonment, due to the prolonged persistence of high decomposition rates inherited from prior tillage disturbances [48]. Meanwhile, wasteland in this study showed no carbon accumulation and maintained a high carbon dissipation pattern consistent with farmland, verifying the widespread occurrence of carbon loss in the early stage of farmland abandonment. This counterintuitive phenomenon is primarily attributed to strong agricultural legacy effects [49]. Wasteland soils exhibited extremely high URE activity, which was attributed to the accumulation of high total nutrients (TP, TK) in the soil background that relieved resource limitation for microbial metabolism [50]. Kuzyakov & Blagodatskaya proposed that residual soil nutrients create microbial hotspots that stimulate intensive extracellular enzyme secretion and trigger strong enzymatic mining of native soil carbon [51]. Our multivariate analysis also revealed that TOC was significantly regulated by URE, providing direct in situ field evidence for this cutting-edge theory. This reveals a critical compensatory mechanism during the early stage of pose-abandonment succession. Legacy agricultural nutrients disrupt the inherent stoichiometric balance. To acquire nitrogen encapsulated in organic matter, microbes accelerate cometabolic processes and secrete hydrolases to degrade the overall SOC structures, promoting intense carbon dissipation. To scavenge endogenous nitrogen, microbes upregulate urease secretion to drive enzymatic mining of exposed organic substrates [46,52]. Although this mechanism sustains ecosystem primary productivity via nitrogen release [53], it also explains the high risk of labile carbon loss in saline soils of the North China Plain in the early abandonment period. Previous studies have demonstrated that litter stoichiometry strongly shapes microbial nutrient acquisition strategies in forest ecosystems. Based on ecoenzymatic stoichiometry and microbial functional decomposition models, high C:N inputs compel microorganisms to regulate extracellular enzyme secretion to alleviate nitrogen limitation [54,55]. Consistent with these findings, our results showed MBC and PRO activity in woodland soils were maintained at a high level and significantly regulated by TN. The high C:N ratio of forest litter induces protease secretion to relieve nitrogen constraints, and the acquired nitrogen further sustains robust microbial biomass with elevated MBC [56]. Continuous turnover of these highly active microbial communities ultimately promotes the accumulation of microbial necromass carbon via the microbial carbon pump (MCP) pathway. Such active assimilation and metabolic processes not only validate the stoichiometric hypotheses from previous research but also provide field-scale empirical support for the microbial carbon pump (MCP) theory proposed by Liang et al. [22].

### 3.2. Physical Protection Mechanisms of Soil Aggregate on Soil Fractions

Soil aggregates are the core unit of physical protection for SOC [31], with their particle size distribution and structural stability directly determining the spatial accessibility of soil organic matter, and thus regulating organic carbon decomposition, turnover rates, and long-term sequestration potential [9,57]. Numerous previous studies have confirmed the positive effect of vegetation root activity on aggregate formation: root entanglement and rhizodeposit binding effectively promote microaggregate adhesion and aggregation, drive microaggregate-to-macroaggregate transformation, and facilitate carbon occlusion [20,31,58]. In this study, we found that different vegetation types in the Hengshui Lake wetland strongly regulate the spatial distribution and turnover of SOC by altering the particle size distribution and stability of soil aggregates. Specifically, reed marsh soils were dominated by macroaggregates, accompanied by high aggregate stability, as well as elevated contents of TOC and POC. These results are highly consistent with previous findings, indicating that the long-term colonization of hydrophytic vegetation can establish a stable physical protection system for SOC by optimizing aggregate structure [20,58]. In addition, regarding the microscopic mechanism underlying aggregate physical protection, previous studies have demonstrated that the physical encapsulation within dense macroaggregates effectively restricts the erosion of core organic carbon sources by hydrolases [47,59]. The exceptional carbon sequestration capacity (high TOC and POC) of reed marsh soils may be attributed, on the one hand, to the physical isolation of organic substrates from microbial and enzymatic decomposition through encapsulation within macroaggregates; on the other hand, the sustained humification process in the anaerobic wetland environment further generates structurally dense humic substances that possess intrinsic chemical recalcitrance [41,42,43]. Consequently, carbon stability in reed marsh habitats is maintained by a dual stabilization mechanism, rather than relying solely on physical entrapment. Our Mantel analysis further showed that soil TOC spatial variation was significantly positively correlated with R_0.25_ (Mantel r = 0.105, *p* = 0.049). Meanwhile, macroaggregates (>0.25 mm) in the 0–10 cm layer of the reed marsh surface soil sequestered the predominant proportion of organic carbon. This result aligns with the viewpoint proposed by Six et al. that macroaggregates serve as the key carrier for short-term soil organic carbon storage [31]. Furthermore, we found that soil aggregate stability was strongly positively correlated with CAT activity, and strongly negatively correlated with PRO activity and EC. The coupling of high EC values with low aggregate stability observed in this study appears to contradict the conventional consensus that salinity can trigger clay flocculation to form aggregate formation [27]. This apparent inconsistency can be fully explained by the concentration-dependent dual effects of soil salinity. Low-level salinity can effectively compress the electric double layer of soil colloids and facilitate clay particle flocculation, which is conducive to the initial formation of microaggregates. As salinity increases, the thickness of the diffuse electric double layer rises, inducing swelling and dispersion of clay particles and consequently triggering the breakdown of soil aggregates [60]. Combined with the strongly alkaline soil environment of the study area (pH > 8.65, Table 2), the co-occurrence of high salinity and strong alkalinity synergistically aggravates the dispersion of soil colloids and the collapse of aggregate structure. Previous studies have also indicated that soil aggregate stability is conversely reduced under high-salinity irrigation conditions [61], which is consistent with the result of the present study. This further complements and validates the “salinity-physical structure-carbon turnover” microscopic mechanism proposed by Rath & Rousk: high salinity stress disrupts soil colloid flocculation equilibrium, triggers soil particle dispersion and aggregate disintegration, and weakens the binding stability between mineral colloids and organic matter; aggregate structure damage exposes large quantities of originally occluded organic fractions, greatly increasing organic matter spatial accessibility and ultimately intensifying microbially mediated carbon decomposition [38]. This also interprets the synergistic degradation effect, in which environmental stress invalidates the physical protection barrier and eventually undermines the stability of soil carbon pools. Our study further clarifies the cascading effect of salt stress–aggregate degradation–accelerated carbon decomposition in inland saline wetland ecosystems. Long-term anthropogenic disturbance has fundamentally reshaped the soil aggregate structure and carbon protection mechanism of farmland and short-term fallow land [31]. At the global macro scale, Sanderman et al. highlighted that long-term agricultural practices disrupt natural soil profiles and result in substantial soil carbon debt [62]. In this study, both farmland and wasteland soils maintained a high R_0.25_ (>85.00%), whereas their internal carbon concentrations were distinctly lower than those in the reed marsh, presenting a contradictory feature characterized by high macroaggregate proportion yet weak carbon sequestration capacity. This finding solidifies the conclusion of widespread agricultural carbon loss induced by anthropogenic interference [62] and further reveals that the agricultural carbon debt and legacy effects persist stably even after short-term farmland abandonment. To elucidate this discrepancy, previous studies have illustrated that long-term tillage reshapes natural soil structure into loose, human-modified aggregates with high internal porosity, where 30–150 μm pores serve as active microhabitats facilitating extracellular enzyme-driven mineralization [63,64]. Consistent with these microstructural mechanisms, our results further confirm that short-term abandonment fails to reverse tillage-induced loose aggregate framework. Furthermore, the lack of structural reconstruction via dense root systems further hinders the recovery of stable soil structure. Accordingly, highly active hydrolases such as protease (PRO) can readily penetrate these aggregates, causing the physically protected internal carbon pool to be largely exposed as labile organic carbon (LOC) and subject to rapid depletion. This field evidence strongly supports and echoes the cutting-edge viewpoint proposed by Lehmann et al. [45], who argued that soil carbon persistence does not depend on passive physical occlusion, but instead relies heavily on constant care through continuous organic matter inputs and natural soil structural reorganization. Nevertheless, this also suggests that in woodland soils lacking physical barrier protection, inherently stable organic fractions are fully exposed and thus subjected to intense enzymatic mining. In contrast to the well-developed physical protection in the reed marsh, the woodland soils exhibited distinctly characteristic in our study. Woodland showed obvious fragmentation of the physical protection barrier provided by macroaggregates, with R_0.25_ below 48.26% and the lowest MWD and GMD. Meanwhile, the SOC content in the >5 mm and 5–2 mm aggregate fractions of the surface layer decreased sharply. Previous studies have proposed that aggregate structure fragmentation and physical barrier degradation significantly increase the “spatial accessibility” of soil organic matter and accelerate organic substrate decomposition and utilization [9,57], a theoretical prediction fully consistent with our woodland results. Meanwhile, our findings align with those of Kothawala et al. [65]: fresh carbon sources directly exposed to aerobic environments promote microbial proliferation and generate abnormally high MBC and DOC. However, this high metabolic state without microstructural protection leads to rapid mineralization and release of highly labile carbon, which is the core limiting factor for TOC accumulation. This finding further reveals the intrinsic differences in how different vegetation communities regulate aggregate structure and thus differentially affect carbon sequestration. From a long-term perspective, intense microbial metabolic activity in woodland ecosystems may drive the chemical transformation of organic molecules and promote the large-scale production of highly condensed aromatic macromolecules with intrinsic chemical recalcitrance through lignin oxidation and other biogeochemical pathways [44]. This implies that, during the late stage of woodland restoration, the continuous operation of the microbial carbon pump (MCP) produces biochemically modified organic components with strong intrinsic chemical stability. These stable fractions can gradually compensate for the lack of physical protection and eventually become the core mechanism sustaining the long-term stability of soil carbon pools in woodland habitats [22,43,44].

### 3.3. Microbial Mechanisms Underlying SOC Fractions Distribution

Soil microorganisms are the core biological engine driving terrestrial ecosystem organic carbon sequestration and turnover [66]. The Microbial Efficiency-Matrix Stabilization (MEMS) framework shows that microbial carbon use efficiency (CUE), together with the microscale physical and mineral protection of metabolic byproducts, jointly determines the ultimate fate of soil organic carbon [21]. Meanwhile, previous studies on belowground interaction have revealed that vegetation succession profoundly alters soil microenvironmental physicochemical properties, including soil water, nutrient, aeration and thermal conditions, thereby directionally reshaping microbial community structure and shifting metabolic trajectories [67,68]. Our multivariate analyses (PCoA, RDA) further confirmed that vegetation type-induced variations in soil physicochemical properties across Hengshui Lake wetland acted as strong driving factors for the directional succession of bacterial (R = 0.5403, *p* = 0.001) and fungal communities (R = 0.68779, *p* = 0.001), ultimately leading to divergent regulatory pathways of SOC dynamics. Existing studies have documented that in natural wetlands, the enrichment of specific oligotrophic taxa (e.g., Chloroflexi) facilitates the accumulation of deep or stable soil carbon by reducing bulk mineralization rates and efficiently utilizing complex carbon sources [15,69]. In this study, driven by high SWC and POC concentrations in the reed marsh, the bacterial phylum Chloroflexi and fungal Rozellomycota were significantly enriched. The resulting low soil mineralization background substantially promoted TOC accumulation, which is consistent with previous research findings [14,69]. Mechanistically, the anaerobic environment formed by long-term flooding inhibits aerobic mineralization and, in combination with the dense physical barrier provided by highly stable aggregates, substantially reduces the accessibility of microorganisms to organic substrates [9]. This dual protective barrier prevents large amounts of plant-derived carbon from thorough microbial assimilation and decomposition, allowing such carbon to be stably sequestered predominantly in the form of POC [47]. Collectively, these results further elucidate the coupled mechanism linking physicochemical properties, microbial community dynamics, and carbon fraction accumulation. In human-disturbed agricultural and abandoned ecosystems, microbial metabolic pathways undergo fundamental shifts. According to the models proposed by Yang et al. [15] and Cotrufo et al. [21], frequent physical disturbance and pulsed inputs of labile substrates can substantially reshape the balance of r/K selection strategies within soil microbial communities. In our study, farmland and short-term abandoned wasteland experienced long-term mechanical tillage with higher BD, which profoundly altered microbial community composition. Meanwhile, Ascomycota, a phylum tolerant to physical disturbance and capable of rapidly responding to pulsed LOC inputs, became overwhelmingly dominant, providing direct in situ evidence for the above theoretical predictions. Previous model simulations have demonstrated that in environments with relatively abundant resources or fragmented soil physical structure, microorganisms tend to adopt a resource allocation strategy that prioritizes respiratory metabolism over carbon assimilation and sequestration, thereby substantially reducing the potential carbon use efficiency (CUE) of the ecosystem [70]. Combining the results of this study and the findings of McLauchlan [71] reveals strong agricultural legacy effects at the early restoration stage. As validated by Laganière et al. [48], long-term tillage in the past thoroughly disrupted the physical encapsulation of LOC. Meanwhile, abundant residual nutrients (phosphorus and potassium) in the topsoil further relieved microbial growth constraints and sustained high metabolic activity. This integrated evidence supports the speculation proposed by Manzoni et al. [70]: microorganisms secrete abundant extracellular enzymes to drive intensive exploitative degradation of exposed soil organic matter, resulting in substantial carbon loss via respiration. This explains the contradictory pattern in farmland and abandoned land, which exhibit a high proportion of LOC but low total organic carbon (TOC) content. Overall, this study confirms that historical anthropogenic disturbance can long-term restrict the carbon sequestration recovery potential of degraded ecosystems [70]. In contrast, the woodland habitats provide a typical case illustrating the trade-off between microbial assimilation efficiency and the loss of physical protection. Early studies found that sufficient plant carbon inputs in woodlands supply broad ecological niches for heterotrophic microorganisms [72]. Furthermore, more studies confirmed that highly complex interactive microbial networks can synergistically enhance potential microbial carbon use efficiency (CUE) [73,74]. In line with previous reports, our results indicated that abundant available carbon (high DOC and MBC contents) in woodland soils fostered high bacterial diversity and triggered the substantial enrichment of dominant taxa such as Proteobacteria. Such a complex metabolic microecosystem represents a transitional state toward efficient microbial assimilation, enabling the rapid transformation of labile plant-derived carbon into microbial biomass. are consistent with these findings: woodland soil dissolved organic carbon (DOC) and microbial biomass carbon (MBC) increased significantly; sufficient labile carbon input drove higher bacterial diversity, enriched eutrophic efficient assimilation taxa such as Proteobacteria, and formed a high-metabolic, strong-assimilation microbial community that efficiently converts plant-derived labile carbon into microbial necromass and biomass carbon. Nevertheless, the microbial carbon pump (MCP) theory proposed by Liang et al. [75] highlights that microbial necromass carbon derived from MCP pathways requires physical encapsulation by fine soil structures to facilitate its transformation into mineral-associated organic matter (MAOM) and long-term carbon sequestration. By integrating the MCP framework [75] with the updated MAOM conceptual model [59], we identified the core bottleneck limiting carbon retention in woodland ecosystems. Owing to the severe loss of multi-scale physical protection provided by macroaggregates in forest soils, large amounts of newly synthesized microbial necromass, though produced efficiently at active biochemical interfaces, remain fully exposed to aerobic conditions prior to stabilization into MAOM. This exposure greatly increases their turnover and mineralization risks, thereby ultimately constraining the cumulative accumulation of soil TOC in woodland ecosystems.

### 3.4. Restoration Strategies of Hengshui Lake Wetlands

Based on the above analysis of the multi-dimensional carbon sequestration mechanisms, this study provides management insight of “hierarchical zoning and habitat-function alignment” for the ecological restoration of inland saline–alkali wetlands. Firstly, for originally degraded habitats or those requiring urgent carbon sequestration, emergent plants such as Phragmites australis should be prioritized. Leveraging the macroaggregate encapsulation driven by their well-developed root systems, combined with “anaerobic-chemical” dual stress induced by high SWC and saline–alkali conditions, rapid physical sequestration and erosion resistance of POC can be achieved [75]. Notably, this carbon pool is extremely sensitive to hydrological fluctuations, and arbitrary drainage must be strictly prohibited to prevent catastrophic carbon loss. Secondly, for woodland restoration areas, the core management focus is to address the deficiency in physical structure [22,31]. Despite the woodlands exhibiting high carbon assimilation potential via the active microbial carbon pump (MCP), structural fragmentation caused by microaggregation leads to severe early turnover losses [75]. Therefore, “constant care” measures (e.g., retaining litter, amending biochar) are essential to artificially accelerate macroaggregate formation [76], thereby providing physical protection for microbial residues against mineralization and ultimately converting potential into a substantial stable carbon pool [22]. Finally, in the short-term abandoned stage of farmland-to-wasteland succession, the core regulatory goal is to curb heterotrophic respiration loss induced by agricultural legacy effects [72]. In practice, mechanical disturbance must be strictly restricted to protect the residual macroaggregate framework [31], and in situ hydrological restoration should be implemented as early as possible. Reconstructing anaerobic microenvironments can block microbial over-nutrition stimulated by residual nutrients [21,70], thereby interrupting the intense dissipation pathway of LOC and gradually reshaping the long-term carbon sequestration network of natural wetland [9,75].

## 4. Materials and Methods

### 4.1. Study Area

The study was conducted in Hengshui Lake National Nature Reserve (37°31′40″–37°41′56″ N, 115°27′50″–115°42′51″ E), Hengshui City, Hebei Province, China. As the only well-preserved inland freshwater wetland ecosystem in North China, the reserve covers a lake area of 75 km^2^, with an east–west length of 22.28 km, north–south width of 18.81 km, and average water depth of 3–4 m. The region has a warm temperate continental monsoon climate, characterized by an annual average temperature of 13.0 °C and annual mean precipitation of 486.6 mm [28].

### 4.2. Sampling Design and Soil Collection

**Figure 7:**
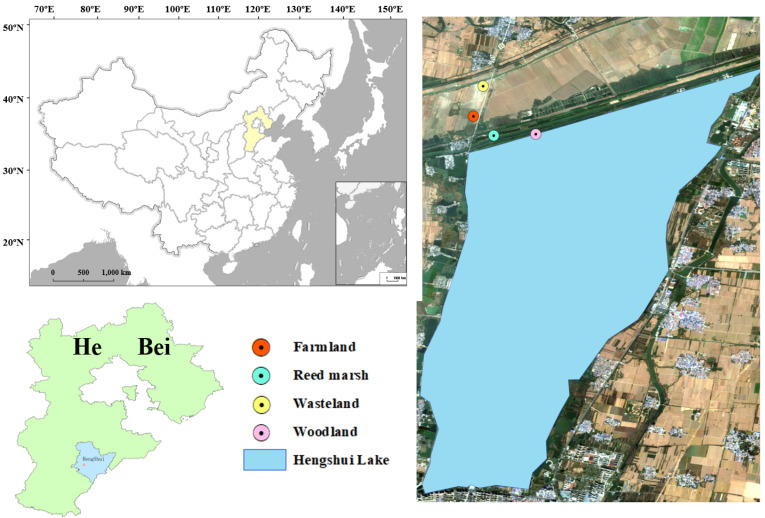
Soil sampling sites in the Hengshui Lake wetland. Four typical vegetation types along Hengshui Lake: Farmland, Reed marsh, Wasteland, and Woodland.

Four typical vegetation types were selected as research sites (Figure 7), including woodland, reed marsh, farmland, and wasteland. The woodland is an artificially planted forest dominated by *Ulmus pumila*, *Koelreuteria paniculata*, and *Robinia pseudoacacia*. The reed marsh is a naturally wetland distributed along the Fudong Drainage Channel, with *Phragmites australis* as the dominant species. The farmland is managed under a wheat–maize rotation system, and maize was the preceding crop before sampling. The wasteland was converted from farmland and is covered in spontaneous vegetation, including *Suaeda salsa* and *Echinochloa crus-galli*.

Soil sampling was carried out in October 2023. For each vegetation type, three repeated plots (separated by ≥100 m to ensure spatial independence) were established, and a 10 m × 10 m quadrat was set in each plot. After removing aboveground biomass and surface litter, undisturbed soil samples were collected at 0–10 cm and 10–20 cm depths using the five-point sampling method. Soil samples from the same quadrat and depth were homogenized into one composite sample, placed in a rigid aluminum box, and transported to the laboratory in a car refrigerator. A total of 24 undisturbed samples (4 vegetation types × 3 replicates × 2 depths) were obtained. Each composite sample was divided into four subsamples for subsequent analyses. The first part was stored at −80 °C for high-throughput sequencing (three technical replicates per sample), the second part was stored at 4 °C for determination of soil MBC and DOC, the third part was air-dried naturally for analysis of soil physicochemical properties and soil enzyme activities, and the fourth part was broken into approximately 1 cm clods along natural fractures, then air-dried for soil aggregate fractionation. Bulk density (BD) was determined using undisturbed soil samples collected with a stainless-steel ring knife (volume = 100 cm^3^).

### 4.3. Analysis of Soil Physicochemical Properties, SOC Fractions and Enzyme Activity

Soil physicochemical properties were determined following standard protocols [77]. pH was determined by potentiometric method with a soil-to-deionized water ratio of 1:2.5 (*w*/*v*) using a pH meter (PHS-3D, Shanghai Leica Instrument Co., Ltd., Shanghai, China). EC was determined using a conductivity meter (DDS-307, Shanghai Precision Instrument Co., Ltd., Shanghai, China) with the same soil-to-water suspension as pH measurement. SWC was determined by oven-drying at 105 °C for 24 h until constant weight. BD was calculated as the ratio of oven-dried soil mass to the ring knife. TN was determined by the Kjeldahl digestion method using an automatic Kjeldahl apparatus (KDN-1, Shanghai Leica Instrument Co., Ltd., Shanghai, China). TP was determined by NaOH alkali melting molybdenum antimony anti colorimetry using a spectrophotometer (TU-1810PC, Beijing Purkinje General Instrument Co., Ltd., Beijing, China), while AP was extracted with NaHCO_3_ and quantified by the same colorimetric method as TP. TK was also determined by NaOH alkali melting followed by flame photometry (ZEEnit 700P, Analytik Jena AG, Jena, Germany). TOC was determined by the H_2_SO_4_-K_2_Cr_2_O_7_ external heating method. MBC was determined by the chloroform fumigation [78]. DOC was determined using a TOC analyzer (multi N/C 2100 TOC, Analytik Jena AG, Jena, Germany) [30]. LOC was determined by the KMnO_4_ oxidation method [79]. POC was determined by using the sodium hexametaphosphate dispersion method [31,69]. Soil enzyme activities were determined following the methods described by Guan [80]. URE activity was determined by the sodium hypochlorite–sodium phenolate colorimetric method, expressed as mg NH_4_^+^-N g^−1^ d^−1^. ALP activity was determined using the disodium phenyl phosphate colorimetric method, expressed as mg p-nitrophenol g^−1^ d^−1^. SUC activity was determined by the 3.5-dinitrosalicylic acid colorimetric method, expressed as mg glucose g^−1^ d^−1^. PRO activity was determined by the casein colorimetric method, expressed as mg tyrosine g^−1^ d^−1^. CAT activity was determined by the KMnO_4_ titration method, expressed as mL 0.1 mol·L^−1^ KMnO_4_ g^−1^ 20 min^−1^.

### 4.4. Soil Aggregate Fractionation and Stability Evaluation

Soil aggregates were separated into six size classes (>5 mm, 5–2 mm, 2–1 mm, 1–0.5 mm, 0.5–0.25 mm, and <0.25 mm) using dry sieving [31]. Briefly, 200 g of air-dried soil was weighed and placed on a set of nested sieves with apertures of 5 mm, 2 mm, 1 mm, 0.5 mm, and 0.25 mm. The nested sieves were mounted on a dry sieve shaker (HF200, Shaoxing Qisheng Instrument Co., Ltd., Shaoxing, China) and shaken at 3000 rpm for 5 min. After sieving, the aggregates remaining on each sieve were collected and weighed to calculate the mass percentage of each size class. A subsample of aggregate from each size class was ground and passed through a 0.25 mm sieve for the determination of intra-aggregate organic carbon (using the H_2_SO_4_-K_2_Cr_2_O_7_ external heating method described in Section 4.3). Soil aggregate stability was evaluated using three indices, R_0.25_, MWD, and GMD [31]. Higher values of R_0.25_, MWD, and GMD indicate greater aggregate stability.

### 4.5. DNA Extraction, PCR Amplification, and High-Throughput Sequencing

Total soil DNA was extracted from 0.50 g of soil samples using the FastPure Soil DNA Isolation Kit (MJYH, Shanghai, China) according to the manufacturer’s instructions. DNA quality and concentration were verified by 1% agarose gel electrophoresis and a NanoDrop2000 spectrophotometer (Thermo Fisher Scientific, Waltham, MA, USA). Qualified DNA samples were stored at −80 °C for four subsequent analyses. The V4–V5 region of the bacterial 16S rRNA gene was amplified using primers 515F (5′-GTGCCAGCMGCCGCGGTAA-3′) and 907R (5′-CCGTCAATTCMTTTRAGTTT-3′) [81]. The ITS1 region of the fungal ITS rRNA gene was amplified using primers ITS1F (5′-CTTGG TCATTTAGAGGAAGTAA-3′) and ITS2R (5′-GCTGCGTTCTTCATCGATGC-3′) [82]. PCR products were separated by 2% agarose gel electrophoresis, purified using the PCR Clean-up kit (YuHua, Shanghai, China) according to the manufacturer’s instructions, and quantified with a Qubit 4.0 Fluorometer (Thermo Fisher Scientific, Waltham, MA, USA). Purified amplicons were pooled in equimolar concentrations and paired-end sequenced on an Illumina Nextseq2000 platform (Illumina, San Diego, CA, USA) by Majorbio Bio-Pharm Technology Co., Ltd. (Shanghai, China) following standard protocols.

### 4.6. Sequencing Data Processing

The raw readings were quality-filtered by fastp version 0.19.6 [83] and merged by FLASH version 1.2.7 [84]. Low-quality reads (e.g., quality score < 20 and read length < 50 bp) were discarded. Above 10 bp overlapped sequences were merged. Finally, 3,173,317 (32,506 to 61,676 sequences per sample) and 5,778,674 (44,263 to 131,687 sequences per sample) high-quality sequences for bacteria and fungi were obtained. Optimized sequences were clustered into operational taxonomic units (OTUs) at 97% sequence similarity using UPARSE v7.1 [85]. Representative sequences of each OTU were annotated against the SILVA database (v 138) for bacteria and UNITE databases (v 8) for fungi using the RDP Classifier with a confidence threshold of 0.7 [86]. Not-targeted OTUs, including OTUs unidentified and assigned as chloroplast and mitochondrion, and mitochondrial sequences were removed. Then, the OTU tables were subsampled (29,506 sequences per sample for the bacterial community and 39,263 sequences per sample for the fungal community for further analysis). All bioinformatic analyses were conducted on the Majorbio Cloud platform (https://cloud.majorbio.com, continuously available online).

### 4.7. Statistical Analysis

Data on SOC fractions, physicochemical properties, enzyme activities, and aggregate stability indices were subjected to one-way analysis of variance (ANOVA) using SAS 9.4 software, followed by the least significant difference (LSD) test for multiple comparisons (*p* < 0.05). At the OUT level, microbial alpha diversity indices (Ace, Chao, Shannon, and Simpson indices) were calculated using Mothur v.1.30.1 [87] to evaluate community richness and diversity, and differences among treatments were analyzed via the Kruskal–Wallis rank-sum test. Relative abundance plots of dominant bacterial and fungal phyla were generated in R v4.5.2. Pairwise correlations among soil environmental factors were assessed using Pearson correlation analysis. Associations between each individual environmental factor and each individual SOC fraction were examined via pairwise Mantel tests: Euclidean distance matrices were constructed for each variable/fraction, and the Mantel test (999 permutations) determined the correlation coefficient (*r*) and significance level (*p*). All environmental variables were standardized to Z-scores pre-analysis to eliminate dimensional effects, with combined network visualization of Mantel test and Pearson correlation analysis results performed in R (v 4.5.2). Principal Coordinate Analysis (PCoA) based on the Bray–Curtis distance was used to evaluate similarities in microbial community structure among treatments (a total of 9 microbial samples per treatment were used, including 3 biological replicates × 3 technical replicates), and analysis of similarities (ANOSIM) was applied to test the significance of inter-treatment differences. Key environmental variables were screened via variance inflation factor (VIF) analysis (VIF < 10) to address collinearity. Redundancy analysis (RDA) was performed to identify key factors shaping microbial communities, and the reliability of explanatory power was verified using the Monte Carlo permutation test (999 permutations). To match the three biological replicates of soil physicochemical data, microbial community data were averaged across technical replicates within each biological replicate, yielding three biological replicates per treatment for RDA. Heatmaps showing correlations between dominant phyla and SOC fractions were constructed based on Spearman rank coefficients. All tables and figures were prepared using Origin Pro 2024, R software (v.4.5.2), and Microsoft Excel 2021.

## 5. Conclusions

This study elucidated the divergent mechanisms of soil organic carbon (SOC) sequestration across four typical vegetation types in inland mild saline wetlands of the North China Plain, highlighting vegetation succession as the key driver regulating soil microenvironments, aggregate physical protection, and microbial metabolic processes. Vegetation-mediated shifts in abiotic conditions and microbial community functions fundamentally shape the accumulation and stability of SOC fractions, leading to distinct carbon sequestration pathways among wetland vegetable types.

Natural reed marshes exhibit the greatest SOC sequestration potential, governed by a coupled abiotic physical protective mechanism. High soil water content and moderate saline–alkali stress form anaerobic, low-decomposition microhabitats, while well-developed macroaggregates provide effective physical occlusion of particulate organic carbon. Unlike conventional freshwater wetlands, salinity-related abiotic constraints override enzymatic regulation of carbon turnover, effectively suppressing microbial mineralization and maintaining stable SOC storage. In contrast, farmland and early abandoned wasteland suffer continuous labile carbon loss due to persistent agricultural legacy effects. Long-term tillage disrupts aggregate structure and anaerobic protection, whilst residual soil nutrients alleviate microbial nutrient limitation and accelerate organic carbon mineralization, resulting in low and unstable SOC pools during early restoration stages. Woodland wetlands rely on a microbial carbon pump-dominated pathway to transform plant-derived labile carbon into microbial necromass under nitrogen-limited conditions. However, fragmented macroaggregate structures fail to provide effective physical protection, causing rapid decomposition of microbially stabilized carbon and limiting net SOC accumulation.

Based on these mechanistic findings, targeted vegetation and habitat management strategies are proposed for regional wetland restoration. Reed vegetation restoration should be prioritized to enhance carbon sequestration via anaerobic and aggregate dual protection. Woodland restoration requires supplementary measures to promote macroaggregate reconstruction and strengthen physical carbon protection. For abandoned farmland, reducing mechanical disturbance and restoring in situ hydrological conditions can effectively mitigate legacy-induced carbon loss and rehabilitate wetland carbon sequestration functions.

## Figures and Tables

**Table 1:** Soil organic carbon fraction distributions under different vegetation types.

Index	Soil Depth (cm)	Woodland	Reed Marsh	Farmland	Wasteland
TOC (g kg^−1^)	0–10	7.81 ± 0.33 b	15.92 ± 1.62 a	7.41 ± 0.08 b	8.71 ± 0.35 b
	10–20	6.69 ± 0.61 a	9.35 ± 2.37 a	8.38 ± 1.05 a	8.49 ± 1.57 a
POC (g kg^−1^)	0–10	4.41 ± 0.74 b	6.65 ± 0.88 a	2.33 ± 0.02 c	1.71 ± 0.41 c
	10–20	1.60 ± 0.22 c	3.26 ± 0.16 a	2.18 ± 0.18 b	1.47 ± 0.15 c
LOC (g kg^−1^)	0–10	1.45 ± 0.23 d	2.06 ± 0.15 c	3.66 ± 0.32 a	2.69 ± 0.26 b
	10–20	0.85 ± 0.09 b	0.88 ± 0.15 b	3.42 ± 0.85 a	2.68 ± 0.36 a
DOC (mg kg^−1^)	0–10	239.75 ± 12.47 a	85.89 ± 2.73 d	155.39 ± 13.61 b	127.23 ± 7.37 c
	10–20	309.39 ± 49.08 a	86.46 ± 2.97 c	164.80 ± 22.20 b	97.44 ± 8.74 c
MBC (mg kg^−1^)	0–10	558.28 ± 33.05 a	451.64 ± 56.99 b	530.21 ± 28.87 a	215.93 ± 15.83 c
	10–20	489.66 ± 64.67 a	233.51 ± 32.59 b	407.54 ± 46.29 a	198.23 ± 33.45 b

Note: Data are expressed as means ± SD. *n* = 3 replicates. Different lowercase letters indicate significant differences among vegetation types within the same soil layer (*p* < 0.05), the same as below. TOC, total organic carbon; POC, particulate organic carbon; LOC, labile organic carbon; DOC, dissolved organic carbon; MBC, microbial biomass carbon.

**Table 2:** Soil physicochemical properties under different vegetation types.

Index	Soil Depth (cm)	Woodland	Reed Marsh	Farmland	Wasteland
SWC (%)	0–10	13.43 ± 0.49 b	44.93 ± 5.19 a	14.73 ± 1.06 b	16.66 ± 0.27 b
	10–20	18.43 ± 2.45 bc	42.05 ± 0.25 a	14.87 ± 2.48 c	23.89 ± 5.48 b
BD (g cm^−3^)	0–10	1.33 ± 0.06 b	0.96 ± 0.10 c	1.58 ± 0.16 a	1.36 ± 0.01 b
	10–20	1.29 ± 0.11 b	1.31 ± 0.03 b	1.55 ± 0.11 a	1.30 ± 0.02 b
pH	0–10	8.71 ± 0.18 a	8.80 ± 0.38 a	8.83 ± 0.11 a	8.65 ± 0.33 a
	10–20	8.73 ± 0.19 a	8.98 ± 0.24 a	9.05 ± 0.04 a	8.84 ± 0.22 a
EC (μs cm^−1^)	0–10	372.94 ± 97.09 a	462.33 ± 54.33 a	91.91 ± 16.44 b	155.20 ± 17.12 b
	10–20	454.56 ± 137.78 a	285.22 ± 21.19 b	86.96 ± 12.712 c	134.47 ± 15.16 c
TN (g kg^−1^)	0–10	0.61 ± 0.03 c	1.30 ± 0.14 a	1.06 ± 0.05 b	1.09 ± 0.19 b
	10–20	0.55 ± 0.12 c	1.39 ± 0.29 a	0.84 ± 0.11 b	0.78 ± 0.05 b
TP (g kg^−1^)	0–10	0.47 ± 0.04 d	0.82 ± 0.01 c	0.92 ± 0.05 b	1.09 ± 0.07 a
	10–20	0.44 ±0.02 d	0.70 ± 0.00 c	0.89 ± 0.05 a	0.81 ± 0.02 b
AP (mg kg^−1^)	0–10	4.33 ±0.86 c	8.40 ± 0.05 b	12.63 ± 1.01 a	11.33 ± 1.46 a
	10–20	4.44 ±0.61 b	4.49 ± 0.29 b	7.25 ± 0.34 a	7.94 ± 0.09 a
TK (g kg^−1^)	0–10	3.12 ±0.27 d	15.51 ± 0.08 b	7.48 ± 0.16 c	17.95 ± 0.87 a
	10–20	4.77 ±0.01 d	15.01 ± 0.25 b	7.68 ± 1.20 c	16.97 ± 0.60 a
	10–20	0.88 ±0.08 c	1.67 ± 0.23 b	2.05 ± 0.03 a	2.01 ± 0.22 a

Note: Different lowercase letters indicate significant differences among vegetation types within the same soil layer (*p* < 0.05). SWC, soil water content; BD, bulk density; EC, electrical conductivity; TN, total nitrogen; TP, total phosphorus; AP, available phosphorus; TK, total potassium.

**Table 3:** Soil enzyme activities under different vegetation types.

Index	Soil Depth (cm)	Woodland	Reed Marsh	Farmland	Wasteland
URE (mg g^−1^ d^−1^)	0–10	0.46 ± 0.03 b	0.53 ± 0.03 b	0.53 ± 0.02 b	1.31 ± 0.46 a
	10–20	0.55 ± 0.01 b	0.11 ± 0.01 d	0.34 ±0.02 c	1.03 ± 0.04 a
PRO (mg g^−1^ d^−1^)	0–10	0.24 ± 0.03 a	0.14 ± 0.01 b	0.17 ± 0.03 b	0.10 ± 0.01 c
	10–20	0.22 ± 0.01 a	0.20 ± 0.03 a	0.14 ± 0.02 b	0.10 ± 0.02 c
ALP (mg g^−1^ d^−1^)	0–10	1.28 ± 0.22 b	1.58 ± 0.12 ab	1.70 ± 0.18 a	1.74 ± 0.20 a
	10–20	1.13 ± 0.13 b	1.26 ± 0.24 ab	1.31 ± 0.18 ab	1.65 ± 0.26 a
SUC (mg g^−1^ d^−1^)	0–10	15.76 ± 1.18 d	35.92 ± 0.18 a	31.72 ± 1.21 b	21.15 ± 1.89 c
	10–20	13.06 ± 1.02 b	9.76 ± 0.27 c	16.66 ± 1.12 a	19.24 ± 2.81 a
CAT (mL g^−1^ 20 min^−1^)	0–10	0.67 ± 0.09 c	1.64 ± 0.09 b	2.29 ± 0.04 a	1.74 ± 0.04 b
	10–20	0.88 ± 0.08 c	1.67 ± 0.23 b	2.05 ± 0.03 a	2.01 ± 0.22 a

Note: Different lowercase letters indicate significant differences among vegetation types within the same soil layer (*p* < 0.05). URE, urease activity; ALP, alkaline phosphatase activity; SUC, sucrase activity; PRO, protease activity; CAT, catalase activity.

## Data Availability

The original contributions presented in this study are included in the article. Further inquiries can be directed to the corresponding authors.
